# A vascular smooth muscle cell X-box binding protein 1 and transglutaminase 2 regulatory circuit limits neointimal hyperplasia

**DOI:** 10.1371/journal.pone.0212235

**Published:** 2019-04-03

**Authors:** Ramon L. Serrano, Weifang Yu, Robert M. Graham, Ru Liu- Bryan, Robert Terkeltaub

**Affiliations:** 1 Department of Medicine, Veterans Affairs Healthcare System, University of California San Diego, California, United States of America; 2 Victor Chang Cardiac Research Institute, Darlinghurst, New South Wales, Australia; Qatar University College of Health Sciences, QATAR

## Abstract

Neointimal hyperplasia, stimulated by injury and certain vascular diseases, promotes artery obstruction and tissue ischemia. In vascular smooth muscle cell (VSMCs), multiple modulators of protein handling machinery regulate intimal hyperplasia. These include elements of the VSMC unfolded protein response to endoplasmic reticulum stress (UPR^ER^), and transglutaminase 2 (TG2), which catalyzes post-translational protein modification. Previous results for deficiency of UPR^ER^-specific mediator XBP1, and of TG2, have been significant, but in multiple instances contradictory, for effects on cultured VSMC function, and, using multiple models, for neointimal hyperplasia *in vivo*. Here, we engineered VSMC-specific deficiency of XBP1, and studied cultured VSMCs, and neointimal hyperplasia in response to carotid artery ligation *in vivo*. Intimal area almost doubled in Xbp1^fl/fl^ SM22α-CRE+ mice 21 days post-ligation. Cultured murine Xbp1 deficient VSMCs migrated more in response to platelet derived growth factor (PDGF) than control VSMCs, and had an increased level of inositol-requiring enzyme 1α (Ire1α), a PDGF receptor-binding UPR^ER^ transmembrane endonuclease whose substrates include XBP1. Cultured XBP1-deficient VSMCs demonstrated decreased levels of TG2 protein, in association with increased TG2 polyubiquitination, but with increased TG transamidation catalytic activity. Moreover, IRE1α, and TG2-specific transamidation cross-links were increased in carotid artery neointima in Xbp1^fl/fl^ SM22α-CRE+ mice. Cultured TG2-deficient VSMCs had decreased XBP1 associated with increased IRE1α, and increased migration in response to PDGF. Neointimal hyperplasia also was significantly increased in Tgm2^fl/fl^ SM22α-CRE+ mice at 21 days after carotid ligation. In conclusion, a VSMC regulatory circuit between XBP1 and TG2 limits neointimal hyperplasia in response to carotid ligation.

## Introduction

Neointimal hyperplasia, characterized by cellular and extracellular matrix expansion, is an arterial phenotype that can be triggered by direct endothelial injury, indirect and disease-associated vessel injury, and disruption of blood flow [1–8]. Specific conditions that can trigger neointimal hyperplasia include shear stress, mechanical (post-revascularization) and hypertensive injury to endothelium, and diseases such as atherosclerosis and inflammatory vasculitis (eg, giant cell arteritis) [1–8]. Significantly, neointimal hyperplasia in response to arterial injury, such as angioplasty, can be rapid and produce marked arterial stenosis or restenosis [9].

Cells involved in neointimal hyperplasia can be activated by agonists including PDGF, angiotensin II, and certain inflammatory cytokines and danger signals [1,4,5,8]. Responses to such mediators, including VSMC synthetic to proliferative differentiation phenotype switch, migration into the intima from the artery media, and apoptosis, play a core role in determining outcome in multiple models of neointimal hyperplasia [1–8]. Important VSMC responses to stress include proteostasis pathways, as well as intersection of elements of the protein handling machinery [1–4,8]. For example, UPR^ER^ is a physiologic response to cell stress due to accumulation of unfolded or misfolded proteins in the ER [10–14]. UPR^ER^ processes include signaling cascades initiated through 3 distinct ER-resident transmembrane proteins [10–15]. Downstream effects include limitation of protein translation, and induction of expression of multiple chaperones. These responses facilitate successful resolution of cell stress, via augmented capacities for protein folding, and degradation of unfolded and misfolded proteins [10–15]. Significantly, excess induction of C/EBP homologous protein (CHOP) in UPR^ER^ promotes neointimal hyperplasia post-injury and atherosclerosis progression, via increased oxidative stress and apoptosis [16,17]. IFNγ, through stimulation of ubiquitin-dependent liver X receptor- degradation, exacerbates neointimal hyperplasia as a result of induction of ER stress and apoptosis in macrophages [8].

Several processes pertinent to intimal hyperplasia are promoted by activation of the transmembrane endonuclease inositol requiring enzyme-1α (IRE1α) pathway of the UPR^ER^ [10,14,15]. These include activation of inflammatory signaling via transmembrane IRE1α-induced phosphorylation of IκB kinase (IKK) and c-Jun N-terminal kinase (JNK) [10,14,15], and direct interaction between IRE1 and PDGFRβ that promotes downstream responses to PDGF [1]. IRE1 plays a mediating role in cultured VSMC proliferation in response to PDGF, as shown by IRE1 siRNA knockdown [1,2]. Injured arterial intima demonstrate the UPR^ER^-specific process of alternative splicing and activation of mRNA of the transcription factor XBP1 by generating the transcriptionally active form of XBP1, termed XBP1s [1]. This process is executed via upstream endonuclease activity of IRE1 [10,14,15]. However, results of 2 prior studies have been contradictory for effects on cultured VSMCs and neointimal hyperplasia of XBP1 [1,2].

The ubiquitously expressed enzyme TG2 has been implicated in intimal hyperplasia [3,4]. TG2 not only cross-links proteins by transamidation, but also modulates the autophagy arm of the proteostasis network, and functions as a protein disulfide isomerase and as a GTPase and GTP-modulated signal transducer [18,19]. Effects of TG2 to promote arterial diseases include mediation of PDGF and β-catenin signaling in VSMCs, and promotion of VSMC proliferation, migration, and phenotypic switching [3,4,20]. Conversely, the capacity of TG2 to inhibit certain cytokines and MMPs, and to interact with fibronectin and transforming growth factor-β, can promotes stabilization of the arterial extracellular matrix [21]. Global knockout of TG2 markedly limited inhibited neointimal hyperplasia in response to carotid artery loop injury (3), but either inhibited or failed to inhibit neointimal hyperplasia in response to carotid artery ligation in two different studies [3,4].

Since both XBP1 and TG2 regulate a variety of cell homeostasis and injury response processes, this study investigated the relationship between XBP1 and TG2 in VSMCs. To simplify our approach to study VSMC responses, we focused on effects of VSMC-specific deficiency of XBP1 and TG2 in neointimal hyperplasia in response to carotid artery ligation, a model that does not involve endothelial denudation [22]. In doing so, we assessed for cross-talk between XBP1 and TG2 in VSMCs. Our results reveal a VSMC regulatory circuit between XBP1 and TG2 that limits neointimal hyperplasia in response to carotid artery ligation.

## Materials and methods

### Materials

Tunicamycin was from Sigma-Aldrich (St. Louis, MO). Enhanced chemiluminescence reagents, 5-(biotinamido)pentylaminewere, horseradish peroxidase (HRP)-conjugated streptavidin, and BCA protein assay reagents were from Thermo Fisher (Carlsbad, CA). DMEM and penicillin/streptomycin were from Thomas Scientific (Swedesboro, NJ), fetal bovine serum was Gemini Bio Products (West Sacramento, CA), and HRP-conjugated goat anti-mouse IgG was from Thermo Fisher. Protein G- HRP conjugated was from Bio-Rad (Hercules, CA). SYBR Green for qPCR assay was obtained from Roche (Indianapolis, IN). Nε-(γ-L-glutamyl)-L-lysine-isopeptide antibody was obtained from Zedira GmbH (Germany) XBP1 siRNA and TG2 siRNA were from Santa Cruz Biotechnologies (Dallas, TX) (sc-38628). All other reagents were from Sigma-Aldrich.

### Plasmids employed

6x His-SUMO-1 {1–97} (Accession number P62165), 6x His-SUMO-2 {1–92} (Accession number CAG46970) or 6x His-SUMO-3 {1–93} (Accession number CAA67897) were kindly provided by Dr. Michael Tatham (University of Dundee, UK). HA-TG2 (human) was cloned into pcDNA3.1 (+) (Life Technologies, Inc.) (EcoR1 and Not1 sites) using the primers (forward 5’-AAACCCGAATTCCTATCCTTACGACGTGCCTGACTACGACATGGCC GAGGAGCTGGTCTTAGAGAG-3’; reverse 5’-AAACCCGCGGCCGCTTAGGCGGG CCAATGATGACATTCCG-3’). *Xbp1s* (mouse) (XBP-1 2: pFLAG.XBP1p.CMV2, plasmid #21833) was from Addgene.org (Cambridge, MA). Control plasmid pFLAG-CMV2 (Cat. # E7033) was from Sigma-Aldrich. Murine *Tgm2* cDNA (Accession BC016492, clone 3256943) purchased from Dharmacon (Lafayette, CO; Cat# MMM1013-202702036).

### Generation of mice lacking XBP1 or TG2 in SMCs (*Xbp1*Δ and *Tgm2*Δ mice)

All animal experiments were reviewed and approved by the San Diego VA Medical Center animal subjects committee. *Xbp1*^fl/fl^ mice with loxP sites flanking exon 2 of *Xbp1*, a gift of Dr. Laurie Glimcher (Harvard Medical School, Boston, MA) [23], were backcrossed with SM22α-cre mice (B6.Cg-Tg(Tagln-cre)1Her/J; Jackson laboratories, cat.# 017491, Bar Harbor, ME) that express α-actin-dependent Cre recombinase activity specific to SMCs [24]. The mice were backcrossed for more than 10 generations onto the C57BL/6 background, were born at a Mendelian ratio and developed normally. To generate mice lacking *Tgm2* (*Tgm2*Δ) in SMCs, *Tgm2*^flox/flox^ mice, possessing *loxP* sites flanking exons 6–8 of *Tgm2* [25] were cross-bred with SM22α-Cre transgenic mice.

### Carotid artery ligation

Eight weeks old mice were anesthetized by intraperitoneal injection of a solution of xylazine (5 mg/kg body weight, AnaSed; Lloyd Laboratories), ketamine (80 mg/kg body weight) and acepromazine maleate (1.2 mg/kg (Boehringer Ingelheim, St. Joseph, MO). We followed a protocol previously described in detail [7]. In brief, the left common carotid artery was ligated near its bifurcation with the use of 6–0 silk, with right carotid artery as uninjured control. At 14 and 21 days after injury, animals were anesthetized and perfused with 5 ml PBS, followed by 5 ml of 4% paraformaldehyde for 3 min. Left and right carotid arteries were excised, further fixed for 16 hours and embedded transversely in paraffin. The entire length of and lesion apex identified. For morphometric analyses, images of Verhoeff-van Gieson elastin-stained cross-sections of left and right carotid arteries (6 micron thickness) were analyzed in a blinded manner using AxionVision microscope (Carl Zeiss, AG Oberkochen, DE), and intimal and medial area calculated [7].

For immunohistochemistry (IHC) analyses, paraffin-embedded ligated carotid artery sections were deparaffinized, followed by digestion with 0.125% Trypsin for antigen retrieval. BSA-blocked slides were incubated with primary antibody overnight at 4°C. We used rabbit anti-Nε-(γ-L-glutamyl)-lysine-isopeptide bond, and anti–total IRE1α (NB100-2324; Novus Biologicals, Littleton, CO) antibodies. Horseradish peroxidase (HRP)-conjugated secondary antibody was from Thermo Fisher (Cat# 878963). Diaminobenzidine was the substrate for colorimetric detection.

### VSMC isolation and culture

To isolate primary mouse VSMCs, aortae from 8 weeks old mice were isolated (from aortic root to bifurcation of the renal arteries) and media was digested, as described (7). VSMC differentiation was verified by staining for smooth muscle -actin (mouse monoclonal antibody; cat#ab7817, Abcam) by immunofluorescence (>95% of cells stained positive for smooth muscle -actin). VSMCs were cultured in complete DMEM medium at 37°C, in 5% CO2. Mouse VSMC line Movas-1 cells (ATCC CRL-2797, Manassas, VA), were transfected with XBP1 siRNA (Santa Cruz Biotechnology, sc-38628) where indicated, and collected at 24 h and 48 h post transfection and lysed in RIPA buffer and cell proteins immunoprecipitated using Dynabeads (Thermo Fisher).

### *In Situ* Tissue Transglutaminase (tTG) activity assay of TG2

For *in situ* TG2 activity measurements, XBP1Δ SMCs were preincubated with 5-(biotinamido)pentylamine (Thermo Fisher), a biotinylated polyamine TG2 substrate that the enzyme can use to post-translationally modify proteins, followed by determination of its incorporation into proteins.[26] We measured absorbance at 492 nm on microplate spectrophotometer. As a negative control, we used *Tgm2*Δ VSMCs treated under identical conditions, and *Xbp1*WT VSMCs served as the positive controls for normal levels of TG2 activity.

### Transwell migration assay

To determine cell proliferation, we used the Invitrogen non-radioactive Cell Proliferation Assay kit, per manufacturer instructions. VSMC migration was assayed, as previously described [7]. In brief, we used a Transwell plate (Cat #3422, 8 μm pore size, Corning, Sunnyvale, CA), with VSMCs plated at 1.0 x 10^6^ cells/ml in DMEM supplemented with 10% heat-inactivated fetal calf serum [7]. Numbers of cells migrated per well, were determined after 48 h treatment with or without PDGF, as described [7]. Images were analyzed using a microscope with digital camera (EVOS).

### SDS-PAGE/Western blot analyses

Primary VSMCs were homogenized and lysed in RIPA buffer supplemented with protease inhibitor tablets (Roche). Protein was separated (10 μg, for each sample) on gradient (4–12%) SDS-PAGE, followed by semi-dry transfer onto PVDF membranes (Millipore, Bedford, MA). Membranes were immunoblotted with primary antibody and the appropriate horseradish peroxidase-conjugated secondary antibody (Bio-Rad). Immunoblots were analyzed by enhanced chemiluminescence technique (Thermo Fisher). Primary antibodies to IRE1α, TG2, GAPDH, SUMO1, SUMO2/3, HA, and K48 ubiquitin were from Cell Signaling Technology (Beverly, MA).

### Quantitative reverse transcription (RT)-PCR

RNA was extracted and isolated from cultured smooth muscle cells, utilizing RNeasy mini kit (Qiagen, Hilden, DE). RNA levels were quantified using a Nanodrop 1000 spectrophotometer (Thermo Fisher); samples with 260 nm/280 nm absorbance ratios >2.0 were used for RT. Equal amounts of total RNA from each sample underwent RT reaction, using Transcriptor High Fidelity cDNA Synthesis Kit (Roche), which includes anchored oligo(dT)_18_ and random hexamer primers. Quantitative RT-PCR was done using a Roche480 Light Cycler and mouse-specific primer sets: XBP1s: XBP1s-F: GAGTCCGCAGCAGGTG; XBP1s-R: GTGTCAGAGTCCATGGGA; XBP1-F: CACCTTCTTGCCTGCTGGAC; XBP1-R: GGGAGCCCTCATATCCACAGT [27]; and TGM2-F: AATGCTCCTATTGGCCTGTACCGT, TGM2-R: AGCCTTGGTAGATGAAGCCCTGTT; GAPDH-F: GGAGCGAGACCCCACTAACA, GAPDH-R: CCTGCTTCACCACCTTCTTGA. Expression of genes of interest was calculated as fold change from control.

### Morphometry

Morphometric analysis was carried out on carotid arteries harvested at 14 and 21 days after ligation. All animals were perfused and fixed under physiological pressure. Images of arteries were analyzed with Axion Vision Software (Carl Zeiss AG, Oberkochen, DE). Additionally, under the assumption that the structures were circular, measurements were used to calculate intimal and medial area.

### SUMOylation assay

To analyze SUMOylation within cells, we used Lipofectamine 2000 to transfect HeLa cells (ATCC CRL-2, Manassas, VA) with plasmids encoding wild-type HA-TGM2 along with either SUMO1, SUMO2 and SUMO3 plasmids respectively. Cells were selected and grown according to a previously described protocol [28]. Cell lysates were immunoprecipitated with a HA-specific affinity matrix gel (Roche, Pleasanton, CA), after which immunoprecipitate was washed in lysis buffer (50 mM Tris-Cl pH 7.5, 150 mM NaCl, 0.1% Triton X-100, 1 mM DTT, 10% glycerol, 10 mM EDTA), followed by elution and proteins resolved by SDS-PAGE and immunoblotted using TG2 antibody, RanGAP antibody (Cell Signal, cat# 36067), and protein G HRP-conjugate (BioRad, Hercules, CA).

### Statistical analyses

All values are expressed as mean ± SD. Statistical differences between WT and mutant mice or cells were analyzed by a Student’s t-test. Morphometric analysis of carotid artery was done by 1-way ANOVA. Differences were considered statistically significant at values of p <0.05. Data for cell culture experiments represent three experiments in triplicates using separate cultures.

## Results

### Increased intimal thickening after carotid artery ligation in mice with VSMC-specific XBP1 deficiency

We generated *Xbp*1Δ mice (*Xbp1* ^fl/fl^SM22α-Cre), in which *Xbp1* exon 2 was efficiently deleted specifically in aortic VSMCs. In parallel, we similarly generated *Tgm*2Δ mice (*Tgm2*^fl/fl^SM22α-Cre). Unlike the case for isolated VSMCs, in the *Xbp1*Δ and *Tgm*2Δ mouse whole tissues studied, there was no gross attenuation of XBP1 or TG2, respectively (S1 Fig).

**Fig 1:**
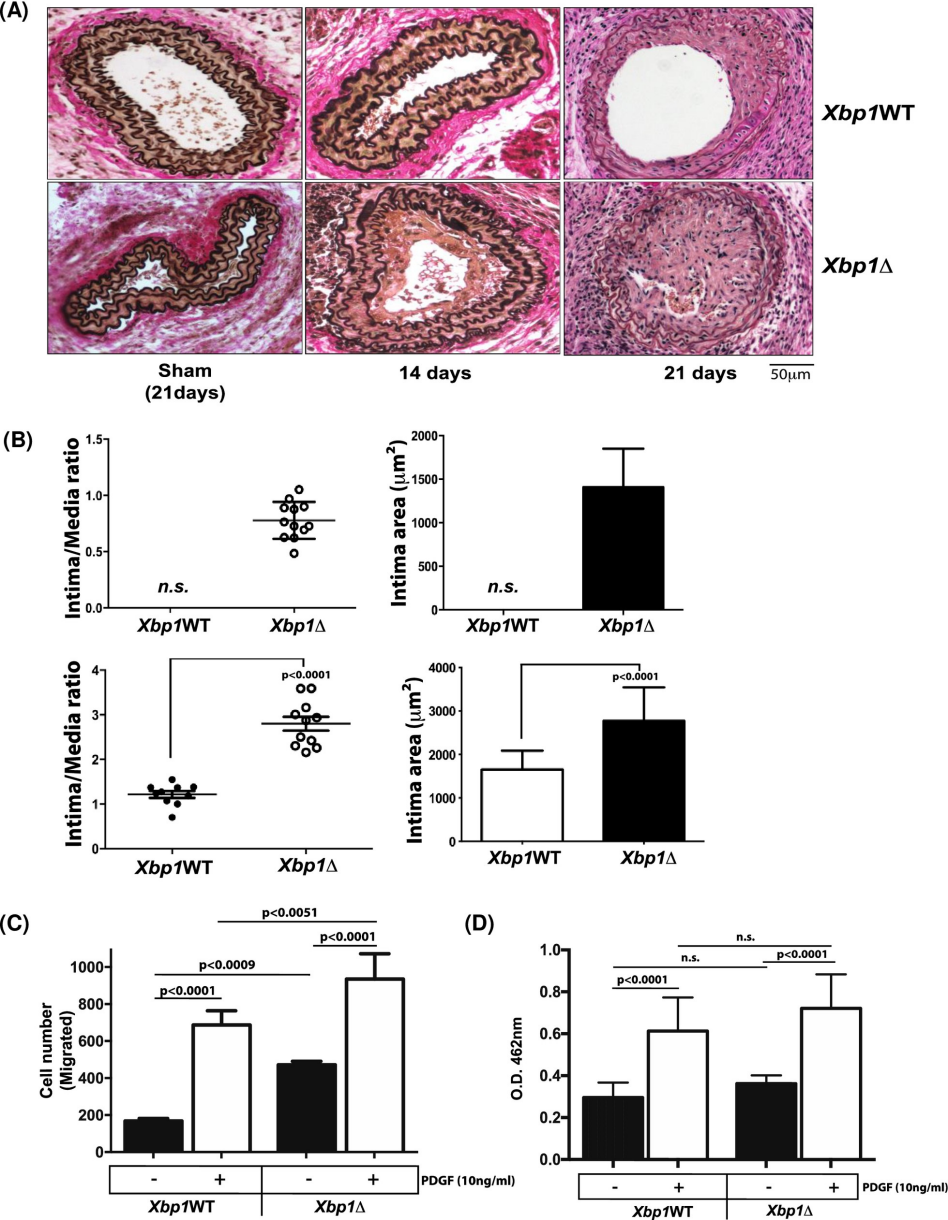
Increased neointimal hyperplasia after carotid artery ligation in *Xbp1* VSMC-specific knockout mice. (A) Mice were studied 14 to 21 days after carotid artery ligation. Verhoff-van Gieson elastin staining of representative sections of *Xbp1*^fl/fl^ (*Xbp1*WT) and *Xbp1l*^*f/fl*^*;SM22*α*-Cre* (*Xbp1*Δ) demonstrated substantial plaque development after 14 and 21 days post ligation. (B) Intima-media ratio was compared for *Xbp1*WT vs *Xbp1*Δ at 14 days post ligation (*p*<0.0001 *n* = 8 *Xbp1*WT; *n* = 12 *Xbp1Δ*), and at 21 days, where intimal cell densities were increased in both *Xbp1*WT and *Xbp1*Δ (*p*<0.0001, unpaired *t*-test, *n* = 10 *Xbp1*WT; *n* = 11 *Xbp1*Δ;). (C) *Xbp1*Δ (*Xbp1*^*f/f*^*;SM22*α*-Cre)* and *Xbp1*WT (*Xbp1*^f/f^) aortic VSMCs were treated with PDGF (10 ng/ml) for 18h. The numbers of cells migrated, and proliferated, were studied as described in the Methods. XBP1 deficiency increased VSMC migration (assessed as the increase in migration in response to PDGF relative to no PDGF) *in vitro* (p<0.0001; *n* = 4). XBP1 deficiency did not significantly increase proliferation in response to PDGF. Data represent mean ± SD of 4 independent experiments. Results were analyzed by 1-way ANOVA, followed by Bonferroni correction).

Neointimal hyperplasia developed in carotid arteries, in response to ligation, in both *Xbp1*WT and *Xbp1*Δ mice. This phenotypic response was significantly increased in *Xbp1*Δ mice (Fig 1A). The lumen of the ligated carotid arteries was larger in *Xbp1*WT mice than in *Xbp1*Δ mice (Fig 1A, whereas intima-media ratio was significantly increased in *Xbp1*Δ mice compared to *Xbp1*WT animals (Fig 1B)). In isolated, cultured aortic VSMCs from *Xbp1Δ* mice treated with PDGF, there was significantly increased migration but not proliferation, compared to control cells (Fig 1C).

### XBP1 modulates TG2 and IRE1α in VSMCs

**Fig 2:**
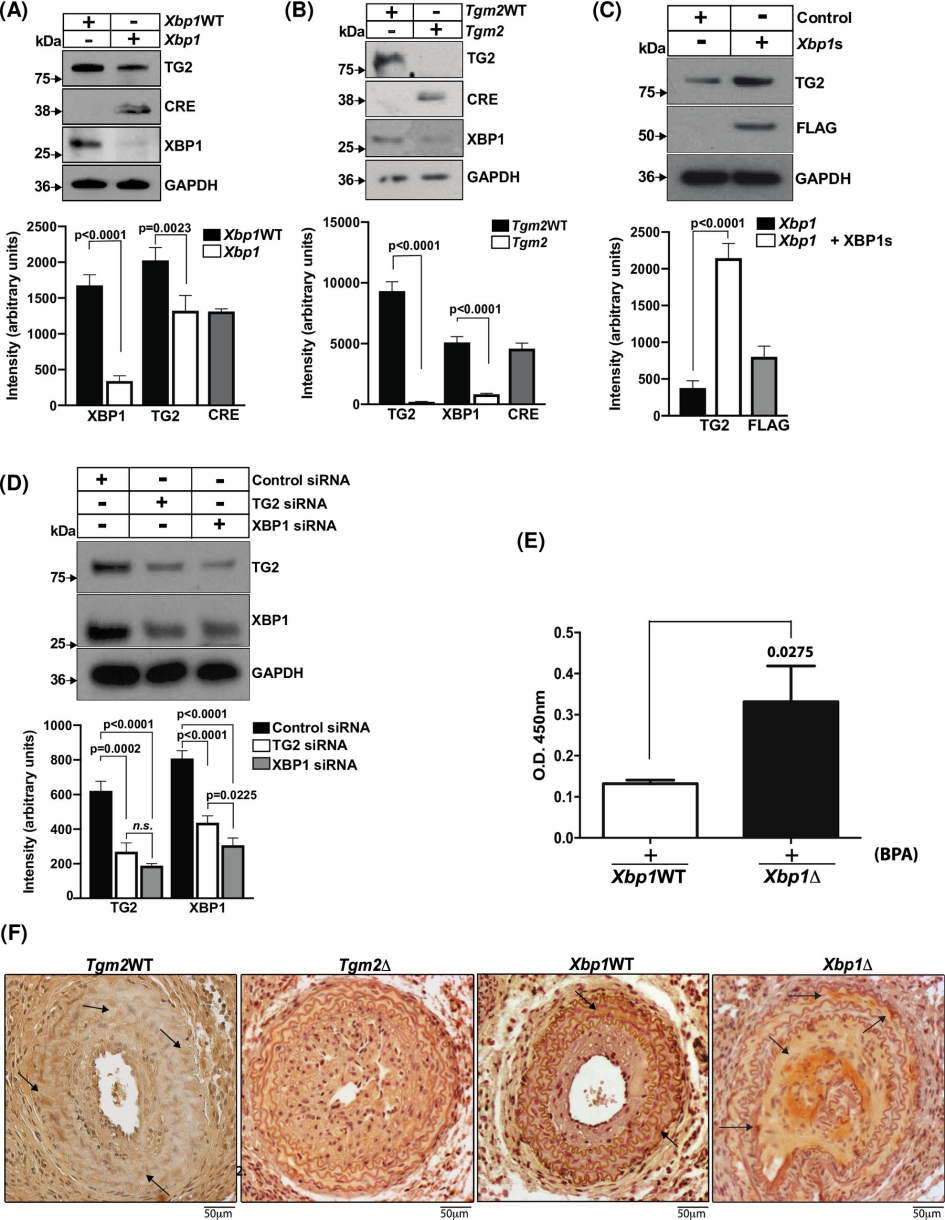
Modulation of endogenous TG2 protein levels by XBP1 in VSMCs. (A) Cell lysates of primary aortic VSMCs from *Xbp1*Δ (*Xbp1*^fl/fl^; SM22α-*Cre*) and *Xbp1*WT (*Xbp1* ^fl/fl^) mice were analyzed by SDS-PAGE-Western blot to determine endogenous levels of TG2, XBP1, Cre recombinase, and GAPDH. TG2 protein levels were significantly higher in WT vs XBP1Δ SMCs with *p*<0.001 (*n* = 4 separate experiments), as determined by densitometry. (B) Cell lysates from primary aortic VSMCs of *Tgm2*Δ (*Tgm2* ^fl/fl^; SM22α-*Cre)* and *Tgm2*WT (*Tgm2* ^fl/fl^) were analyzed by Western blotting to determine levels of TG2, XBP1, Cre recombinase and GAPDH. Absence of TG2 was noted in the *Tgm2*Δ cells. (C) Representative Western blot of lysates of *Xbp1*Δ VSMCs transfected with *Xbp1s* or control plasmid (Unpaired t-test *p* = <0.0001; *n* = 4 separate experiments) showed increased TG2 protein with *Xbp1* transfection. (D) MOVAS-1 cell XBP1 siRNA or TGM2 siRNA knockdown induced decrease in both TG2 and XBP1 protein levels (One-way ANOVA) (E) *In situ* TG2 (tTG) activity in *Xbp1* WT and *Xbp1*Δ VSMCs, determined by incorporation of 5-(biotinamido)pentylamine into protein, showed increased TG2 activity in the *Xbp1*Δ cells. Data were analyzed using Student’s t-test. (F) Carotid artery sections for *Tgm2*Δ, *Tgm2* WT, *Xbp1*Δ and *Xbp1*WT were probed with antibody against Nε-(γ-L-glutamyl)-lysine-isopeptide bond. Representative results of IHC from studies of 3 separate animals of each genotype showed enhanced TG2-mediated crosslinking activity in *Xbp1*Δ sections compared to *Xbp1*WT.

Isolated VSMCs from cultured XBP1 deficient mouse aortae demonstrated decreased TG2 (Fig 2A). Conversely, TG2-deficient VSMCs demonstrated decrease in XBP1u (Fig 2A). Transfection of *XBP1s* into XBP1-deficient VSMCs significantly increased TG2 protein (Fig 2C and inset). Silencing of either *XBP1* or *TGM2* expression by siRNA in MOVAS-1 cells affected the protein expression level of the other protein (Fig 2D). Paradoxically, XBP1 deficiency *in vitro* was associated with increased activity of the TG2 present, as assessed by tTG assay [29](Fig 2E). In response to carotid ligation, the carotid artery sections, analyzed by IHC using an antibody to Nε-(γ-L-glutamyl)-lysine-isopeptide bond, showed increase of the selective TG2 crosslinking marker in *Xbp1*Δ carotid arteries compared to wild type (Fig 2F).

**Fig 3:**
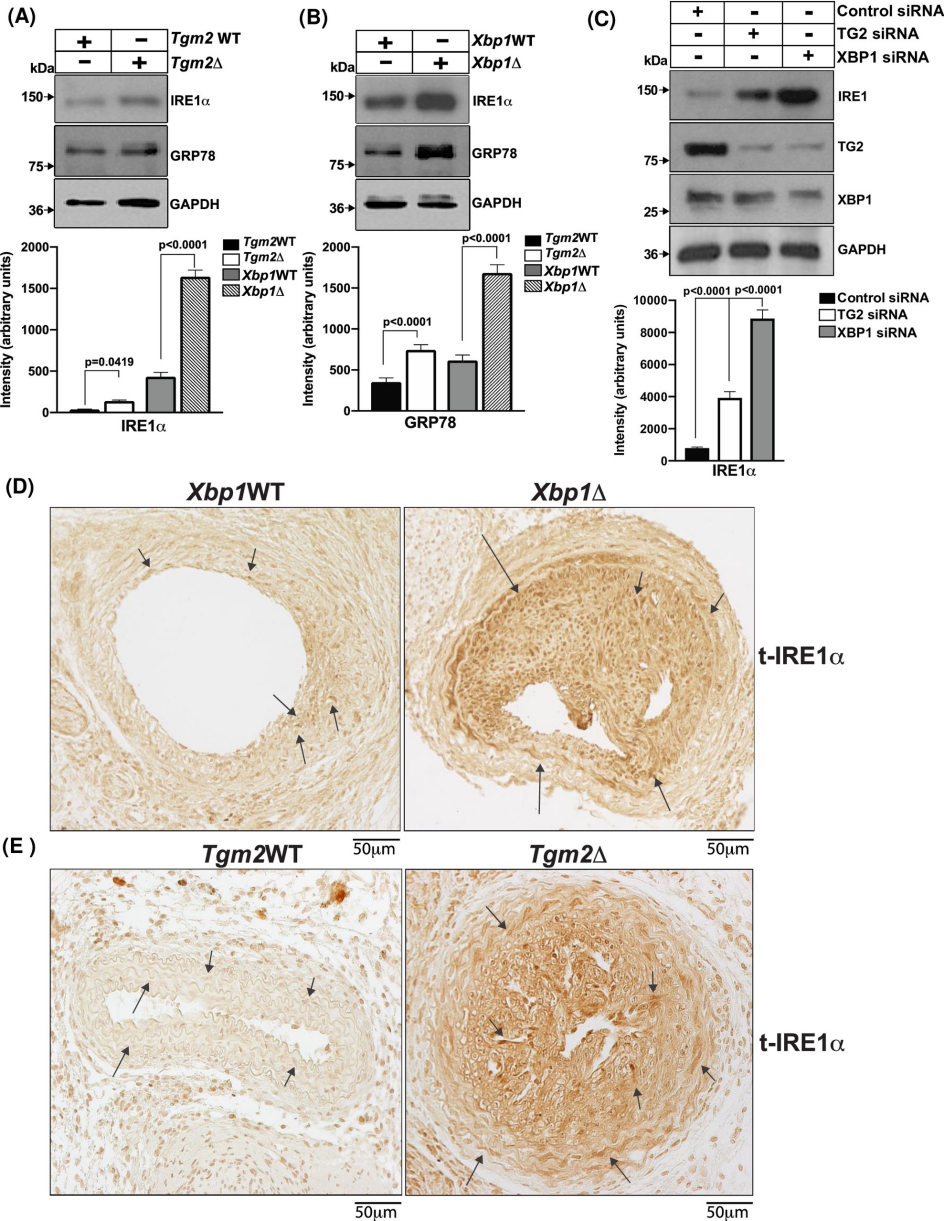
XBP1 and TG2 deficiency promote increased IRE1α protein levels in cultured VSMCs and in neointima *in situ*. (A) Cell lysate protein (10 μg aliquots) from cultured primary *Xbp1*WT and *Xbp1*Δ aortic VSMCs, and *Tgm2* WT and *Tgm2*Δ VSMCs (B) were analyzed for IRE1α, with XBP1, TG2, and GAPDH as internal controls, using SDS-PAGE/Western blotting (Densitometry analyzed by Ordinary one-way ANOVA; n = 4) (C) MOVAS-1 cells transfected with either XBP1 siRNA or TG2 siRNA were probed for IRE1α. The Figure shows representative results (n = 4 separate experiments, p<0.0001, Ordinary one-way ANOVA) for SDS-PAGE/Western blotting of cell lysates using antibodies against IRE1α, with TG2, XBP1, and GAPDH as internal controls. (D) *Xbp1*WT and *Xbp1*Δ WT and (E) *Tgm2* WT and *Tgm2*Δ carotid artery sections were analyzed by IHC for total IRE1α.

IRE1α has multiple binding partners, and many effects on cell differentiation and function independent of action on XBP1 [10,14,15,30,31]. Hence, we assessed IRE1α levels *in vitro* and *in situ*. In VSMCs extracted from aortae of the conditional knockout mice, we observed increased IRE1α levels in *Xbp1*Δ, and also, though to a lesser degree, more IRE1α in *Tgm2*Δ cells (Fig 3B). Similarly, IRE1α was increased in MOVAS-1 cells in which *Xbp1* was knocked down by siRNA (Fig 3C). Moreover, we observed markedly increased IRE1α immune staining in neointima *in situ* in carotid artery sections of XBP1Δ mice (Fig 3D). The mRNA levels for *Tgm2* and *Xbp1* did not significantly decrease with deficiency of the other protein in cultured VSMCs (S2 Fig). Thus, we next tested for effects of XBP1 deficiency on the stability of TG2.

### XBP1 affects TG2 stability in VSMCs

**Fig 4:**
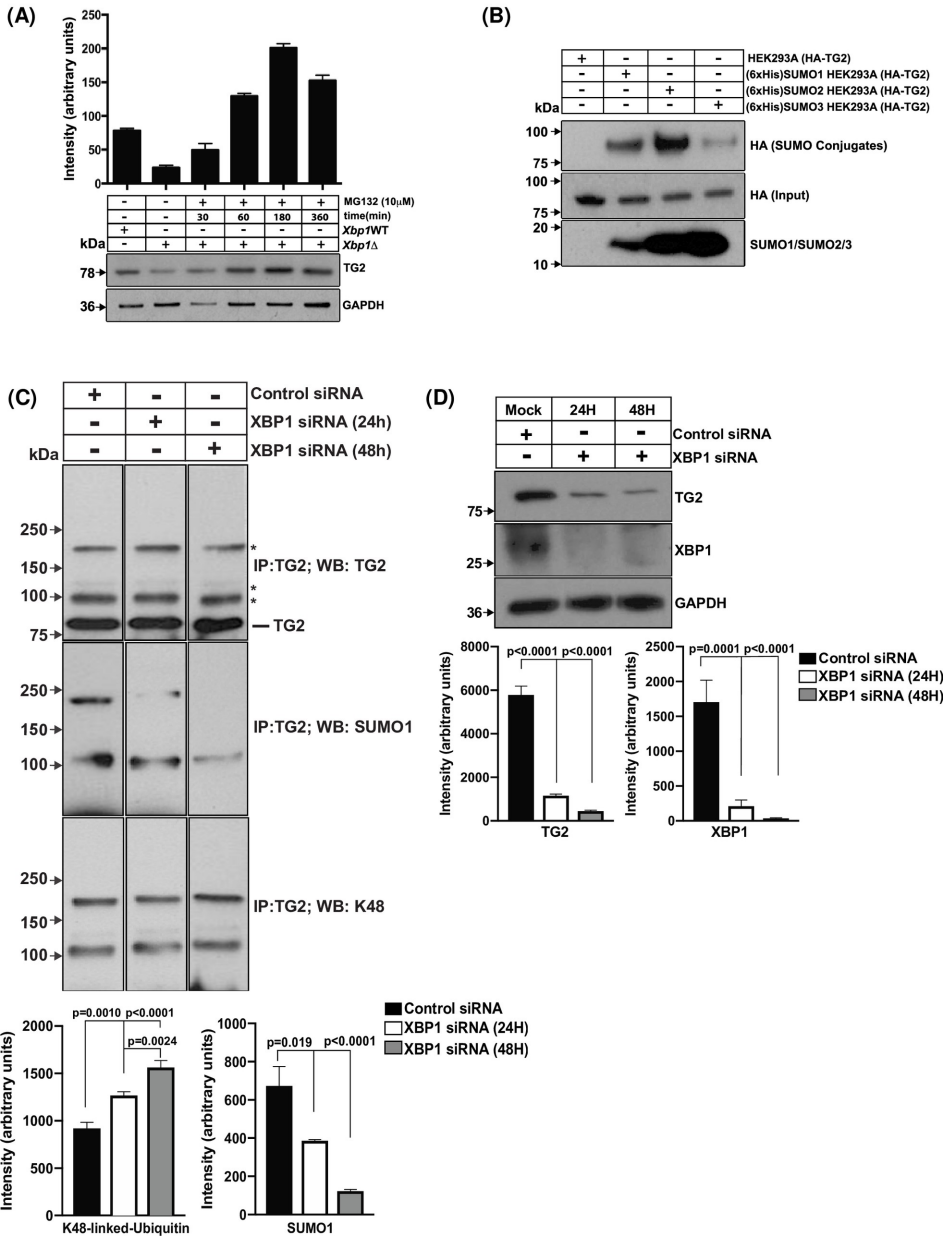
XBP1 deficiency in VSMCs affects TG2 SUMOylation and ubiquitylation. (A) *Xbp1*Δ VSMCs were treated with the proteasome inhibitor MG132 over 6 h, and samples collected at 30, 60, 180 and 360 minutes during this time period. At each time point, cell lysate samples were analyzed by probing Western blots with TG2-specific antibody. (B) HeLa stable lines expressing SUMO proteins, (SUMO1, SUMO2 and SUMO3) and transfected with human HA-*TGM2* construct, were immunoprecipitated using HA-TAG magnetic beads. The eluates were probed using anti-HA antibody. (C) MOVAS-1 cells were transfected with *Xbp1* siRNA, followed by harvesting at 24 h and 48 h thereafter. Homogenates (100 μg aliquots) were immunoprecipitated using TG2-specific antibody and eluates analyzed by Western blots using antibodies to TG2, K48-ubiquitin, and SUMO1. (D) The effect of Xbp1 siRNA knockdown on the level of TG2 in MOVAS-1 cells is shown in the Figure (Densitometric Analysis, n = 3, Ordinary one-way ANOVA).

TG2 levels significantly increased in Xbp1Δ VSMCs treated over time with the proteasome inhibitor MG132 (Fig 4A). Since XBP1 deficiency appeared to promote increased proteasomal TG2 degradation, we next studied TG2 SUMOylation. We also assessed if TG2 cellular stability was potentially subject to dynamics between TG2 SUMOylation and ubiquitylation [32]. To do so, we generated stable HeLa cell lines expressing distinct SUMO molecules (SUMO1, SUMO2 and SUMO3) [33,34], and we transfected these cells with TG2-HA expression plasmid. We first confirmed [32] that TG2 was SUMOylated by SUMO1, (Fig 4B), and predominantly modified by SUMO2 and to lesser extent by SUMO3. The SUMOylation was compared to control RanGAP SUMOylation (S3 Fig). To test whether TG2 ubiquitylation/SUMOylation dynamics can be affected by XBP1 deficiency, MOVAS-1 cells were transfected with XBP1 siRNA, followed by immunoprecipitation at different post transfection times. TG2 demonstrated reduced SUMOylation (SUMO1) in response to *Xbp1* knockdown, and, conversely, there was increased TG2 K48-linked ubiquitylation (Fig 4C), which is known to promote proteasomal degradation [35].

### Increased intimal thickening after carotid artery ligation in TGM2Δ mice

**Fig 5:**
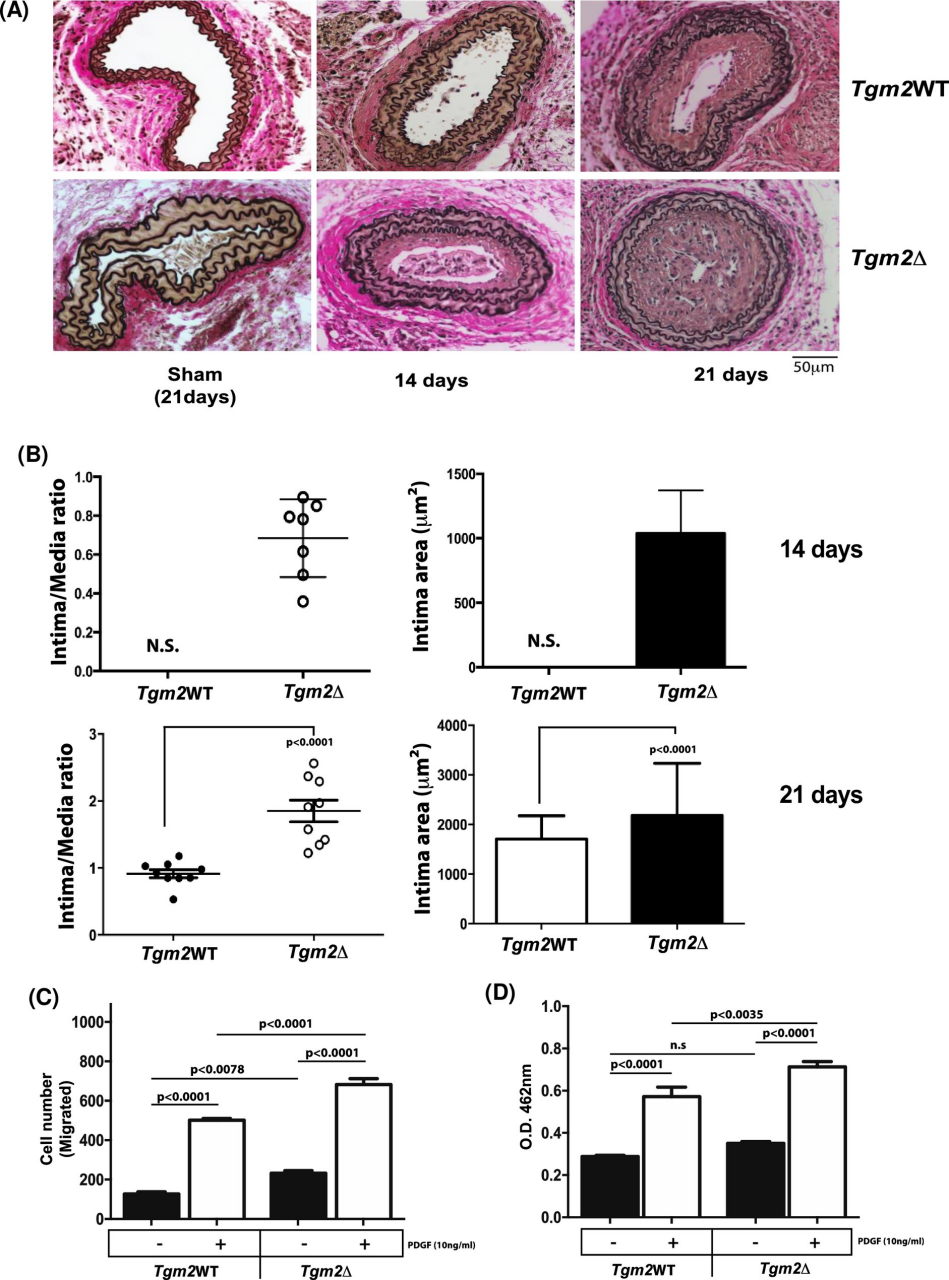
Increased intimal thickening after carotid artery ligation in *Tgm2* VSMC-specific knockout mice. (A) Verhoff-van Gieson elastin staining of representative carotid artery sections from *Tgm2*WT (*Tgm2*^f/f^) and *Tgm2*Δ (*Tgm2*^f/f^;SM22α-*Cre*) mice demonstrated neointimal hyperplasia at 14 and 21 days after vessel ligation. (B) Intima-media ratio was compared for *Tgm2*WT vs *Tgm2*Δ after 14 days after ligation surgery (*p*<0.0001 *n* = 9 WT; *n* = 9 *Tgm2*^f/f^). At 21 days after ligation, cell densities in the intimal lesions in *Tgm2*WT and *Tgm2*Δ were compared (*p*<0.0001, unpaired t-test, *n* = 9 TGM2WT; *n* = 12 TGM2Δ;). (C) *Tgm2*Δ and *Tgm2*^WT^ VSMCs were treated with PDGF (10 ng/ml) for 18 to assess migration and proliferation, as described above. VSMC migration and proliferation were increased in VSMCs *in vitro* (*p*<0.0001; *n* = 9). Data represent mean ± SD of 4 independent experiments. Results were analyzed by 1-way ANOVA, followed by Bonferroni correction).

Neointimal hyperplasia and associated lumen occlusion developed in the ligated carotid arteries of both *Tgm2*WT and *Tgm2*Δ mice, and these responses were significantly increased in *Tgm2*Δ mice (Fig 5A and 5B). Last, isolated *Tgm2*Δ aortic VSMCs demonstrated both increased migration and proliferation in response to PDGF compared to *Tgm2*WT (Fig 5C).

## Discussion

VSMC responses to stress are modulated by the UPR, other proteostasis pathways, and by the multifunctional molecule TG2, a component of the protein handling machinery [1–4,8]. This study identified a regulatory circuit in VSMCs via cross-talk between TG2 and XBP1, a transcription factor whose activation by IRE1 endonuclease action is a UPR-specific event [11–14]. Specifically, deficiency of XBP1 or TG2 promoted decrease in the level of the other respective protein in VSMCs. These changes were not associated with corresponding alteration of XBP1 or TGM2 mRNA levels due to deficiency of TG2 or XBP1, respectively. Instead, we demonstrated an increased capacity of XBP1-deficient cells to promote TG2 proteasomal degradation by increase in K48 polyubiquitylation relative to SUMOylation. Protein SUMOylation has the capacity to promote protein stabilization, in part by blocking ubiquitination at lysine residues, and partly because SUMOylated proteins have a distinct overall surface charge distribution compared to ubiquitin [33,34]. XBP1 was previously recognized to regulate ER-associated protein degradation and to have major effects on the transcriptional profile of the proteostasis network, and on successful UPR resolution [12,13]. These new findings on regulation of TG2 by XBP1 suggest additional means by which XBP1 can modulate cell fate and inflammatory responses following activation of the IRE1 arm of the UPR [10–12,36–38].

VSMCs, as well as endothelial, vascular precursor, and inflammatory cells, and platelets have been implicated in neointimal hyperplasia [1–8]. Arterial wire injury, employed in several of these studies, is designed to resemble post-revascularization injury in stenotic human arteries [22]. However, endothelial injury, and substantial roles of inflammatory cell influx and activation in such murine models of neointimal hyperplasia, render arterial wire injury mechanistically and cellularly more complex than carotid artery ligation [22]. The carotid ligation model is triggered by marked reduction of vessel diameter and attenuation of blood flow, with consequent effects on arterial wall shear stress stimulating rapid VSMC-enriched neointima formation [22]. Our study focused squarely on VSMC biology via unique use of newly generated SMC-specific knockout mice for *Xbp1* and *Tgm2*, and we examined only neointimal hyperplasia following carotid artery ligation.

We demonstrated that both SMC-specific XBP1 and TG2 deficiency increased neointimal hyperplasia in response to carotid artery ligation *in vivo*. In complementary *in vitro* studies here, VSMCs isolated from aortae of both these conditional knockout animals were observed to migrate more in response to PDGF. Furthermore, TG2-deficient VSMCs proliferated more in response to PDGF than did VSMCs from control animals. Here, as in prior studies involving XBP1 and TG2 [1–4], results for cultured VSMC responses to PDGF, and for neointimal hyperplasia were largely aligned. However, multiple differences existed between the conclusions of prior studies VSMC XBP1 function *in vitro* and in neointimal hyperplasia *in vivo* [1,2], now magnified by results of our study. In one prior report, global *Xbp1* haploinsufficiency increased neointimal hyperplasia in response to femoral artery wire injury, and XBP1 siRNA augmented cultured human coronary VSMC migration and proliferation in response to PDGF [2]. In contrast, SM22α-targeted SMC-specific *Xbp1* deficiency increased neointimal hyperplasia in response to femoral artery wire injury in another prior report [1]. Overexpression XBP1s increased human VSMC migration, and conversely, cell migration out of aortic ring explants was decreased by *Xbp1* knockout in one study [2]. However, XBP1 deficiency in cultured VSMCs failed to significantly alter PDGF-induced migration in the same report [2].

Differences in results from small animal neointimal hyperplasia models partly reflect distinctions in experimental model behavior between species, but also expose differences between pathogeneses of models [22]. It is possible that factors in discrepancies between findings on XBP1 in VSMC behavior and neointimal hyperplasia include differences in degree of experimental depression of XBP1 levels, and impact of distinct protocols and reagents and agonists, such as using VEGF or PDGF as agonist. Also involved may be beneficial effects downstream of XBP1 in stressed cells, such as modulation of apoptosis and autophagy [11–13], successful resolution of proteotoxic cell stress, and prevention of oxidative stress by modulating reactive oxygen species generation and catalase expression [39]. Conversely, XBP1s promotes activation of VSMCs via PI3K/Akt signaling and the capacity to down-regulate calponin h1 through mir-1274B-mediated mRNA degradation [1]. Increases in VSMC PDGF and PDGF receptor levels in XBP1-deficient VSMCs [2], and in IRE1α levels as observed here, also merit consideration. IRE1α endonuclease activity is critical to induce XBP1 activation by generating alternatively spliced XBP1s transcript [12,13], but IRE1α also can enhance downstream effects of PDGF receptor signaling [10,14,15]. Moreover, IRE1α endonuclease and kinase activities have multiple additional consequences on cell differentiation and function independent of action on XBP1 [10,14,15]. These include activation of inflammatory signaling and cytokine expression via transmembrane IRE1α -induced phosphorylation of IKK and JNK [12–15], and direct interaction between IRE1 and PDGFRβ that promotes downstream responses to PDGF [1].

TG2 not only has been observed to increase VSMC migration and proliferation *in vitro* [3,4], but also to promote inward remodeling of injured arteries *in vivo* [40]. Moreover, previous work observed significant decreases, in global TG2 knockout mice, of neointima formation in both PDGF-treated heart slices and in carotid arteries following ligation [3,4]. Significantly, prior study reported attenuated neointimal hyperplasia following carotid artery loop injury in global TG2 knockout mice, though there no significant difference between global TG2 knockout and wild type mice in neointimal hyperplasia following carotid artery ligation [3]. As such, our finding that SMC-specific TG2 deficiency increased neointimal hyperplasia, in response to carotid artery ligation, could be viewed as paradoxical. However, we did observe that XBP1 deficiency was associated with increased VSMC TG activity *in vitro*, and neointimal TG2 protein cross-links in response to carotid artery ligation *in vivo*. Hence, our results suggest a model in which increased transamidation activity of TG2, and potentially reciprocal decrease in GTPase-related TG2 activity, as well as decrease in TG2 levels and non-enzymatic activities, could be among the mechanisms whereby stressed XBP1-deficient VSMCs can promote neointimal hyperplasia.

Limitations of this study include that it did not specifically examine effects of VSMC XBP1 and TG2 deficiencies on the extracellular matrix synthesis and composition, or on cell viability, and on the global proteostasis network. *Tgm2* deficient VSMCs had a proliferative response to PDGF-BB (10 ng/ml). However, fleshing out individual factors mediating the migration response in TG2-deficient VSMCs was beyond the scope of this study. It will be of interest to test for potential contributory effects of mitochondrial function regulation by the IRE-XBP1 signaling axis [41] in arterial VSMC responses to vascular injury. It remains to be determined if the VSMC XBP1 and TG2 regulatory loop defined here has generalized impact across intimal hyperplasia disease models, including restenosis after direct endothelial denudation injury.

We conclude that cross-talk between XBP1 and TG2 in VSMCs is substantially involved in limiting the extent of neointimal hyperplasia in carotid arteries injured by ligation. Our findings identify how dysregulated UPR and protein handling machinery responses can not only be intertwined but also amplify adverse adaptive responses to tissue injury. The results provide further support for the potential of systemically and locally controlling the UPR and TG2 to productively intervene in the phenotype of tissue responses to injury.

## Supporting information

S1 Checklist ARRIVEGuidelines%20Checklistillable%29%20copyd.pdf: The ARRIVE Guidelines checklist is attached. (PDF)

S1 Fig Levels of XBP1 and TG2 were not attenuated in selected whole tissues from respective conditional knockout mice. (A) Aliquots of 10 μg of homogenized whole tissues, extracted from *Xbp1* WT and *Xbp1*Δ animals, and (B) *Tgm2* WT and *Tgm2*Δ animals, were analyzed by SDS-PAGE/Western blotting to assess levels of XBP1 and TG2 protein, respectively.
(TIF)

S2 Fig The mRNA levels for *Tgm2* and *Xbp1* were not suppressed by deficiency of the other respective molecule. For quantification by qPCR of *Tgm*2 and *Xbp1* in murine VSMCs, we reverse-transcribed 500 ng of total RNA extracted from cultured smooth muscle cells (VSMCs). We used qPCR to determine mRNA levels of *Tgm2* and *Xbp1* in *Tgm2*WT, *Tgm2*Δ, *Xbp1*WT and *Xbp1*Δ.
(TIF)

S3 Fig SUMOylated proteins in homogenates prepared from stable HeLa cell lines expressing SUMO proteins. For affinity purification of SUMO proteins, we subjected eluates from Nickel-NTA beads to SDS/PAGE-Western blotting, using TG2 and RanGAP as controls for protein SUMOylation. The figure shows anti-TG2, RanGAP1 and input immunoblots of nickel affinity purifications from HeLa cells. The high molecular weight species were detected by TG2 antibody, with strong signal above ~75kD representing TG2 eluted from Ni-NTA columns. Endogenous TG2 was covalently conjugated to 6His-SUMO1, 2 and 3, with eluates compared from stable lines expressing SUMO1,2, and 3. RanGAP antibody was able to detect endogenous protein which is modified by SUMO1, and to lesser extent by SUMO3, and was used as control for the procedure. Non-specific bands are indicated by asterisks.
(TIF)

S1 Dataset Sminimaldataset.xlsx: The minimal data set, for the manuscript results presented here, is attached. (XLSX)
