# Supplementary figures and images for: A vascular smooth muscle cell X-box binding protein 1 and transglutaminase 2 regulatory circuit limits neointimal hyperplasia
Source: PLoS One. 2019 Apr 3;14(4):e0212235. doi: 10.1371/journal.pone.0212235 (PMC6447169; doi:10.1371/journal.pone.0212235)

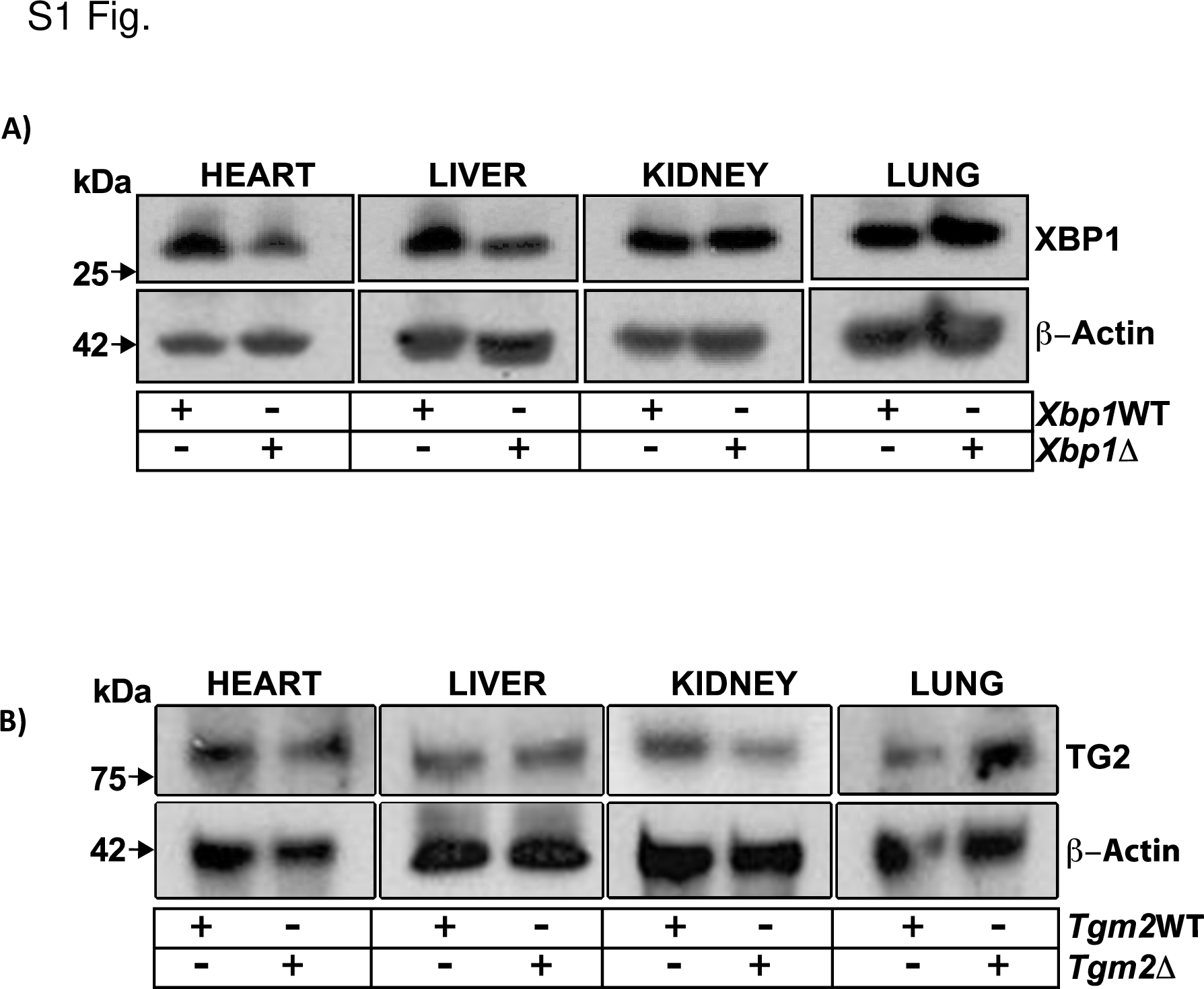

Supplement: S1 Fig — (A) Aliquots of 10 μg of homogenized whole tissues, extracted from Xbp1 WT and Xbp1Δ animals, and (B) Tgm2 WT and Tgm2Δ animals, were analyzed by SDS-PAGE/Western blotting to assess levels of XBP1 and TG2 protein, respectively. (TIF) [file pone.0212235.s002.tif]

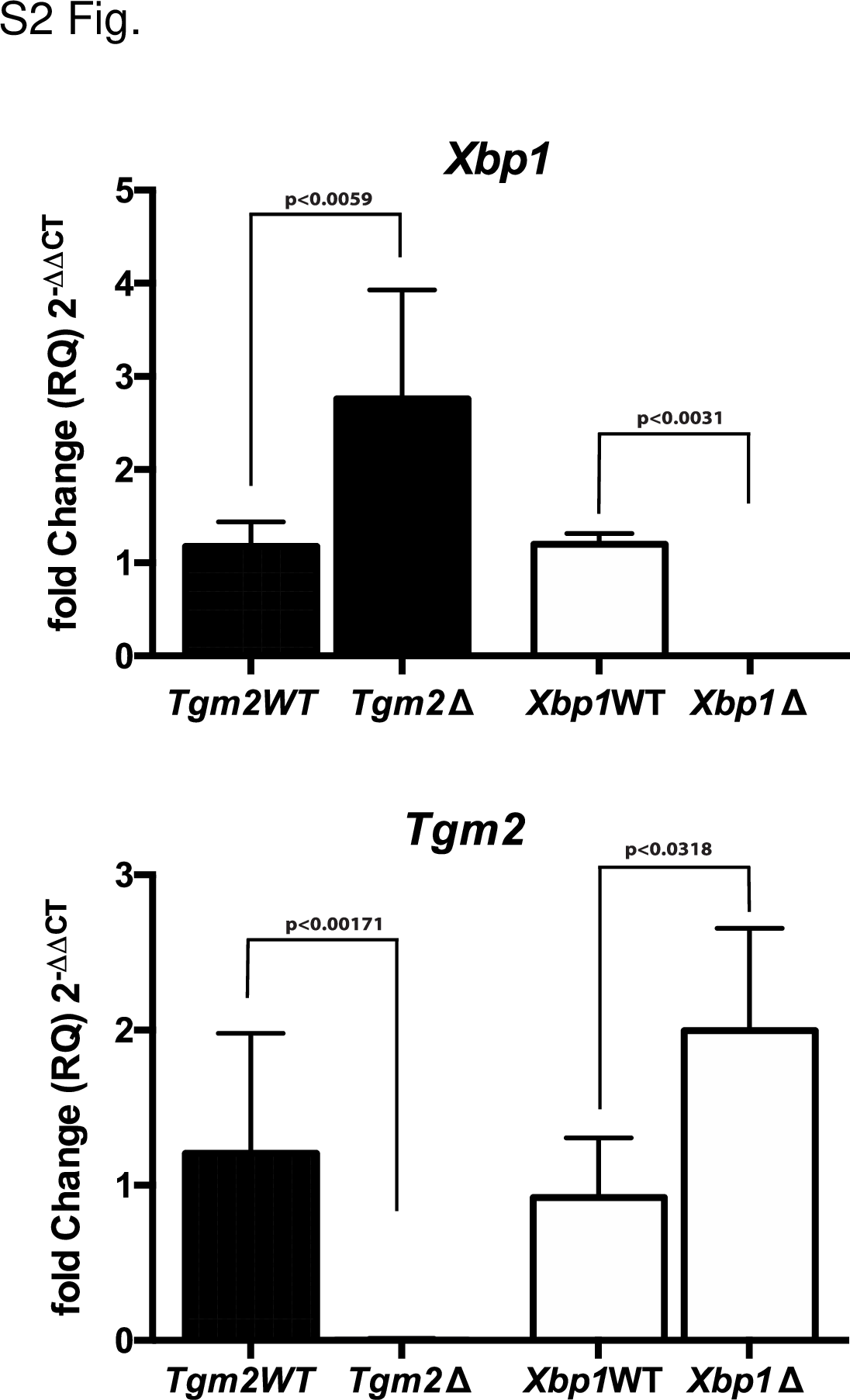

Supplement: S2 Fig — For quantification by qPCR of Tgm2 and Xbp1 in murine VSMCs, we reverse-transcribed 500 ng of total RNA extracted from cultured smooth muscle cells (VSMCs). We used qPCR to determine mRNA levels of Tgm2 and Xbp1 in Tgm2WT, Tgm2Δ, Xbp1WT and Xbp1Δ. (TIF) [file pone.0212235.s003.tif]

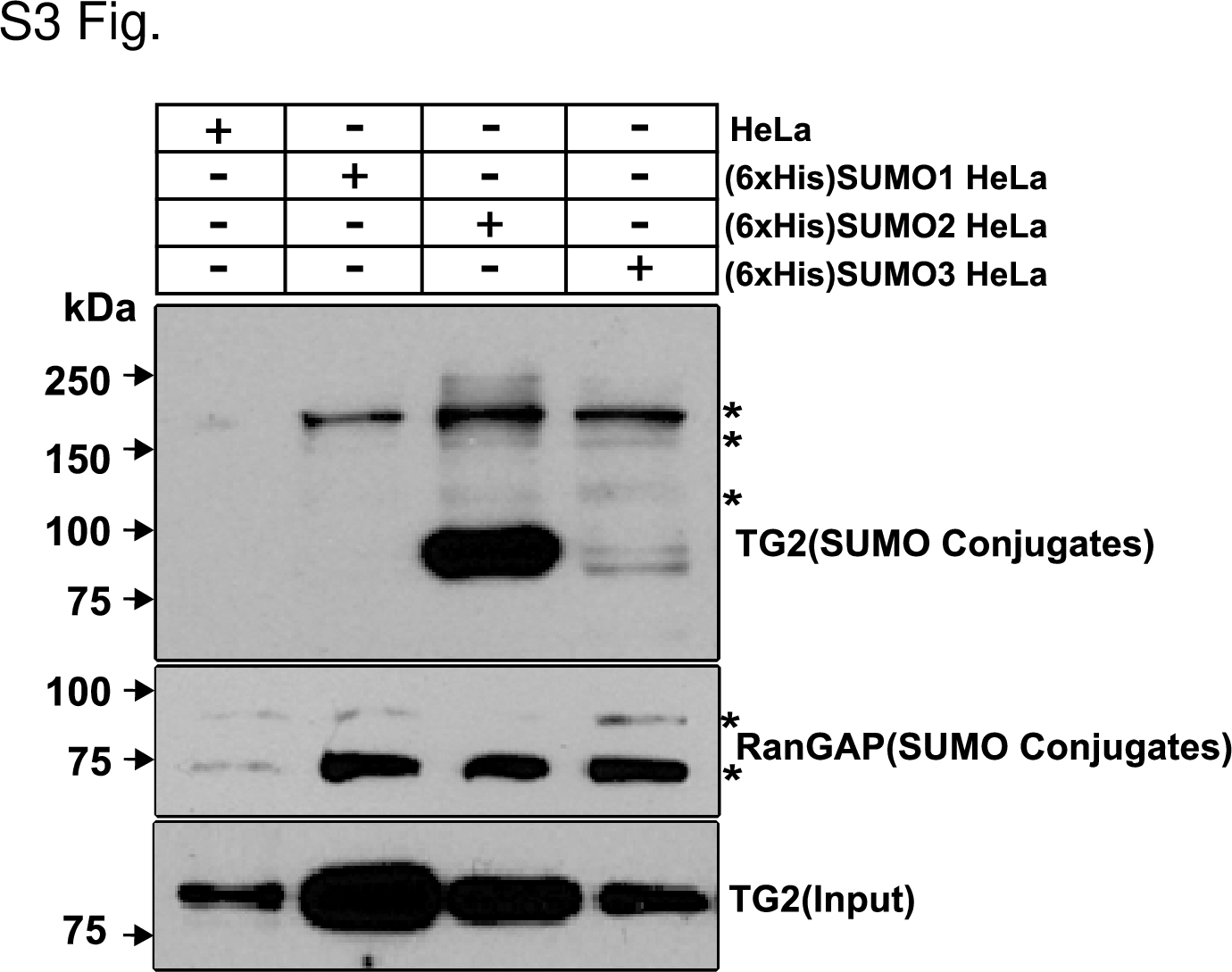

Supplement: S3 Fig — For affinity purification of SUMO proteins, we subjected eluates from Nickel-NTA beads to SDS/PAGE-Western blotting, using TG2 and RanGAP as controls for protein SUMOylation. The figure shows anti-TG2, RanGAP1 and input immunoblots of nickel affinity purifications from HeLa cells. The high molecular weight species were detected by TG2 antibody, with strong signal above ~75kD representing TG2 eluted from Ni-NTA columns. Endogenous TG2 was covalently conjugated to 6His-SUMO1, 2 and 3, with eluates compared from stable lines expressing SUMO1,2, and 3. RanGAP antibody was able to detect endogenous protein which is modified by SUMO1, and to lesser extent by SUMO3, and was used as control for the procedure. Non-specific bands are indicated by asterisks. (TIF) [file pone.0212235.s004.tif]
